# Cleidocranial dysplasia: Radiological mimic of pyknodysostosis – A case report

**DOI:** 10.4102/sajr.v22i1.1326

**Published:** 2018-06-14

**Authors:** Harmeet Kaur, Kamini Gupta, Punit Tiwari

**Affiliations:** 1 Department of Radiodiagnosis, Dayanand Medical College & Hospital, India; 2 Department of Orthopedics, Government Medical College, India

## Abstract

Cleidocranial dysplasia (CCD) is a rare autosomal dominant skeletal disorder with predominant membranous bone involvement. It may also occur as a sporadic mutation. The diagnosis of this condition is based on the clinical, radiological and genetic findings. It is characterised by hypoplasia or aplasia of the lateral thirds of the clavicles; craniofacial and dental anomalies; and hypoplastic iliac bones. Pyknodysostosis is a close radiological mimic of this entity. Definite diagnosis is based on the genetic analysis. A 36-year-old short-statured female was referred for computed tomography of the paranasal regions for complaints of a deviated nasal septum and midline depression in her forehead. Skeletal screening demonstrated an open metopic suture, wormian bones, maxillary hypoplasia, maldentition and aplastic lateral thirds of both clavicles. In this article, we report a case of CCD, discuss various overlapping features between CCD and pyknodysostosis and attempt to differentiate them radiologically.

## Introduction

Cleidocranial dysplasia (CCD) is a rare (incidence of 1:100 000),^1^ autosomal dominant skeletal disorder; however, 40% of cases occur spontaneously with no apparent genetic cause.^2^ It primarily affects bones which undergo intramembranous ossification, but enchondral bones are also affected. The pathology relating to this condition is owing to an early developmental disorder of mesenchyme or connective tissue. This causes retarded ossification of bone precursors, especially at junctions, which can lead to defective ossification, or even failure of ossification of portions of the skeletal structure.^3^

In contrast, pyknodysostosis is an autosomal recessive disorder of osteoclast dysfunction, causing generalised osteosclerosis with associated maxillofacial anomalies. Pyknodysostosis shares many features with CCD.^4^ In this article, we discuss various imaging features that are common to these entities as well as those which help to differentiate them radiologically. A dysplastic flexed clivus with convexity towards the endocranium is characteristic of CCD when present and may be useful in differentiating the two entities.^5^

## Clinical details

A 36-year-old, short-statured female presented to our department for computed tomography (CT) of the paranasal (PNS) region before corrective facial surgery for a deviated nasal septum and midline depression in her forehead. Her birth history was normal. Her intelligence was normal. On physical examination, her weight was 48 kg and height was 120 cm.

She had a midline depression on her forehead which was likely owing to an open metopic suture. Additional findings included a high-arched palate, depressed nasal bridge, maldentition and mandibular prognathism. She was wearing a dental prosthesis. Her shoulders were hypermobile. Her family history was unremarkable, suggesting that her disorder was without any obvious genetic cause.

Based on these findings, craniofacial dysplasia was considered. In addition to CT imaging, a skeletal survey, including radiographs of the skull, shoulders, hands and pelvis, was done.

Laboratory investigations for serum calcium, phosphorus, alkaline phosphatase, parathyroid hormone, thyroid function and vitamin D levels were obtained. They were all within normal range.

**FIGURE 1:**
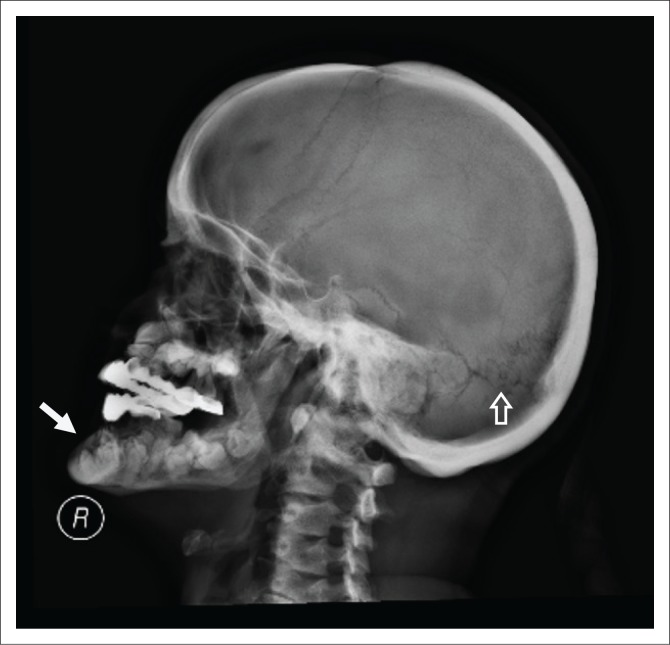
Lateral radiograph of the skull shows normal bone density with wormian bones in the lambdoid suture (hollow arrow), prognathism and supernumerary teeth (white arrow). Patient is wearing dental prosthesis.

**FIGURE 2:**
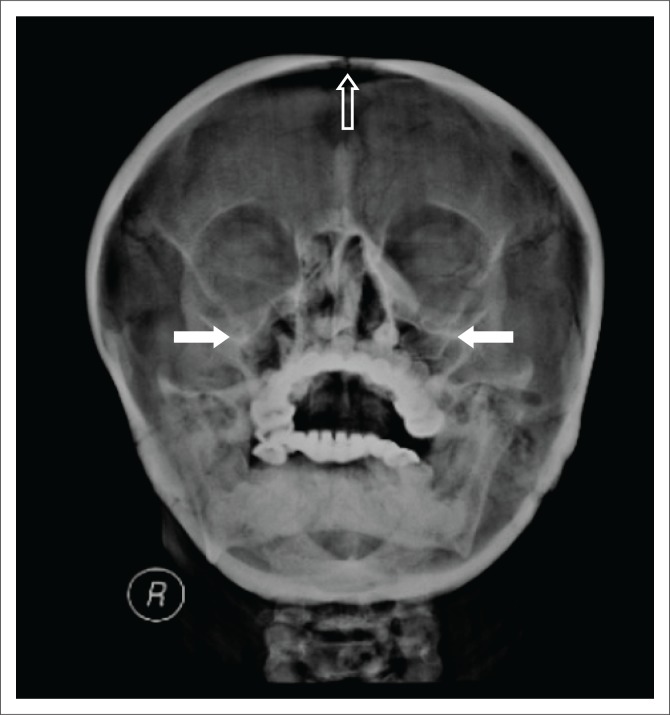
Water’s view for paranasal sinuses shows an open metopic suture (hollow arrow), aplastic frontal sinuses, hypoplastic maxillary sinuses (white arrows) and underpneumatised mastoids.

**FIGURE 3:**
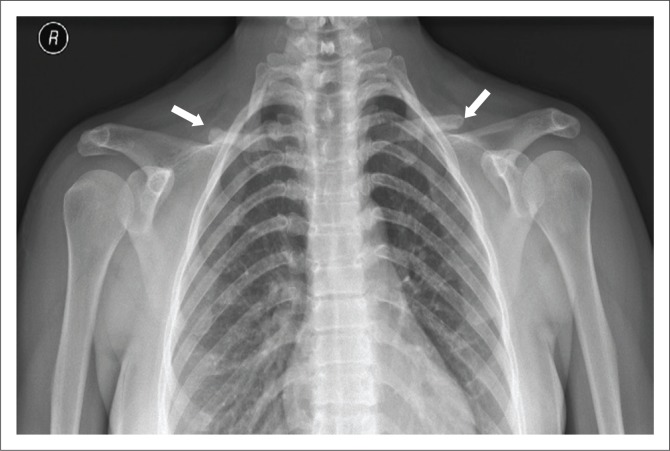
Postero-anterior radiograph of the upper chest shows aplasia of lateral two-thirds of both clavicles (white arrows) and a bell-shaped thorax.

A lateral radiograph of the skull (Figure 1) showed normal bone density, wormian bones (white arrow), mandibular prognathism and supernumerary teeth. Radiographs of the PNS sinuses (Figure 2) revealed an open metopic suture, aplasia of the frontal sinuses, hypoplasia of the maxillary sinuses and underpneumatised mastoids. Radiographs of the shoulders (Figure 3) demonstrated aplasia of the lateral two-thirds of both clavicles and a bell-shaped thorax. Hand and pelvis radiographs were normal.

**FIGURE 4:**
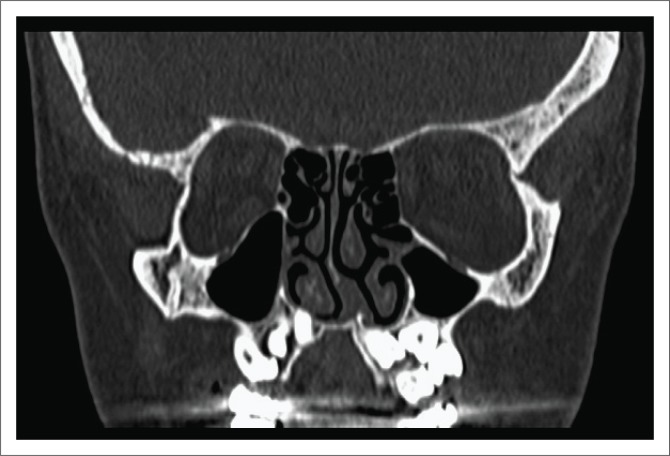
Coronal section of computed tomography-paranasal region shows supernumerary unerupted teeth, a deviated nasal septum, hypoplastic maxillary sinuses and a high-arched palate.

**FIGURE 5:**
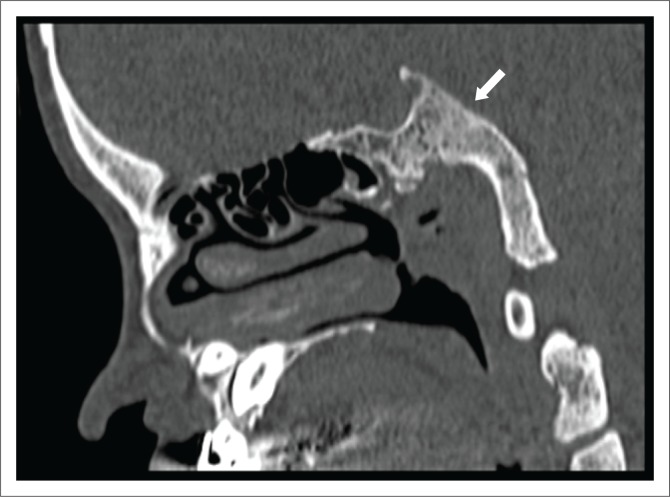
Sagittal-section computed tomography scan shows a dysplastic clivus with its flexion deformity and convexity towards the endocranium (arrow).

**FIGURE 6:**
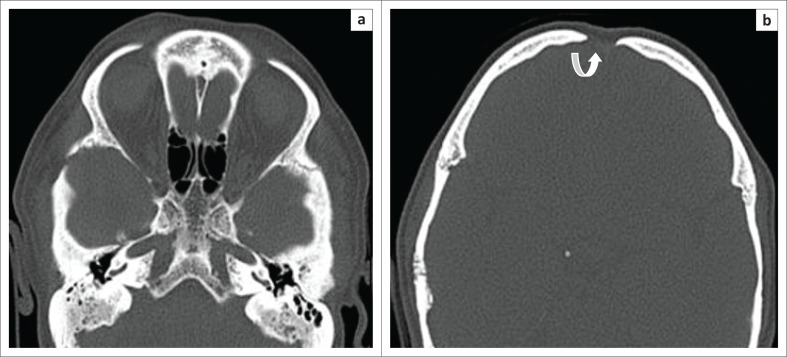
Axial-computed tomography section at the level of skull base (a) shows under-pneumatised mastoids, non-pneumatised sphenoid sinuses and superiorly (b) shows an open metopic suture (curved arrow).

Non-contrast CT of the PNS revealed normal bone density, multiple supernumerary teeth, a deviated nasal septum and a high-arched palate (Figure 4), a dysplastic clivus which was flexed with convexity towards the endocranium (Figure 5), an open metopic suture and sclerotic mastoids (Figure 6). Based on the laboratory and radiological findings, the patient was diagnosed as a case of CCD.

## Discussion

Cleidocranial dysplasia is a skeletal dysplasia with characteristic clinical findings and autosomal dominant inheritance. Typical clinical findings are hypoplastic or aplastic clavicles, abnormal craniofacial growth, supernumerary teeth, short stature and a variety of other skeletal changes. According to our observations and those of Mundlos,^6^ a complete absence of the clavicles is rare, whereas hypoplasia of the acromial end is common. Head circumference is usually at the upper limit without being macrocephalic. There is a broad forehead with frontal bossing and some degree of hypertelorism. The mid-frontal area is poorly developed and shows a frontal groove owing to incomplete ossification of the metopic suture.^6^

Many patients with facial dysmorphism and hypoplastic or aplastic clavicles have gone through life without any functional disability and are detected incidentally. As in our case, the patient was unaware of her disease and was pursuing corrective surgery for cosmetic reasons. But as these diseases have genetic consequences, their exact diagnosis and patient counselling are important. The inheritance of CCD and pyknodysostosis can be variable in a small percentage of cases; hence, their radiological differentiation is also important.

Short stature, frontal bossing and excessive mobility of shoulder girdle are the most significant clinical findings of CCD.^7^

Broad sutures, large fontanelles persisting into adulthood, numerous wormian bones, numerous unerupted deciduous and supernumerary teeth, maxillary hypoplasia with relative mandibular prognathism and hypoplastic or aplastic clavicles are the pathognomonic radiological findings of CCD. Vertebral defects with scoliosis, kyphosis or lordosis; wide pubic symphysis; and anomalies of phalangeal, tarsal, metatarsal, carpal and metacarpal bones may also be present.^8^

Individuals with CCD may have osteopenia and may develop osteoporosis, a condition that makes bones prone to fracture.

Pyknodysostosis is a rare defect of osteoclast function, resulting in cranio-facial dysplasia like CCD. It shares many features with CCD such as, short stature, delayed closure of sutures and fontanelles, wormian bones, supernumerary teeth, hypoplastic PNS sinuses, mandibular prognathism and hypoplasia or aplasia of the acromial ends of the clavicles.

While many of the clinical and radiological findings of both entities resemble each other, the increased radiographic bone density should readily distinguish between the two.^6^ Thus, osteosclerosis (increased bone density resulting in fractures) and acro-osteolytic dysplasia of the distal phalanges are considered essentially pathognomonic of pyknodysostosis.

To our best knowledge, skull base changes in pyknodysostosis include thickening and sclerosis of the clivus with platybasia and basilar invagination. However, a dysplastic flexed clivus with convexity towards the endocranium has not been mentioned in pyknodysostosis till date and is classical of CCD, seen in 82% of cases,^5^ and demonstrated in our case.

## Conclusion

Cleidocranial dysplasia and pyknodysostosis are rare skeletal dysplastic conditions. Although genetic typing is the gold standard, these entities can be diagnosed clinically and radiologically. The pathognomonic feature to distinguish between them is bone density, but we suggest that distortion of the clivus with a flexed deformity in CCD is a useful adjunctive feature to differentiate the two. The importance of recognition of these features in the diagnosis and prevention of future complications is stressed.
